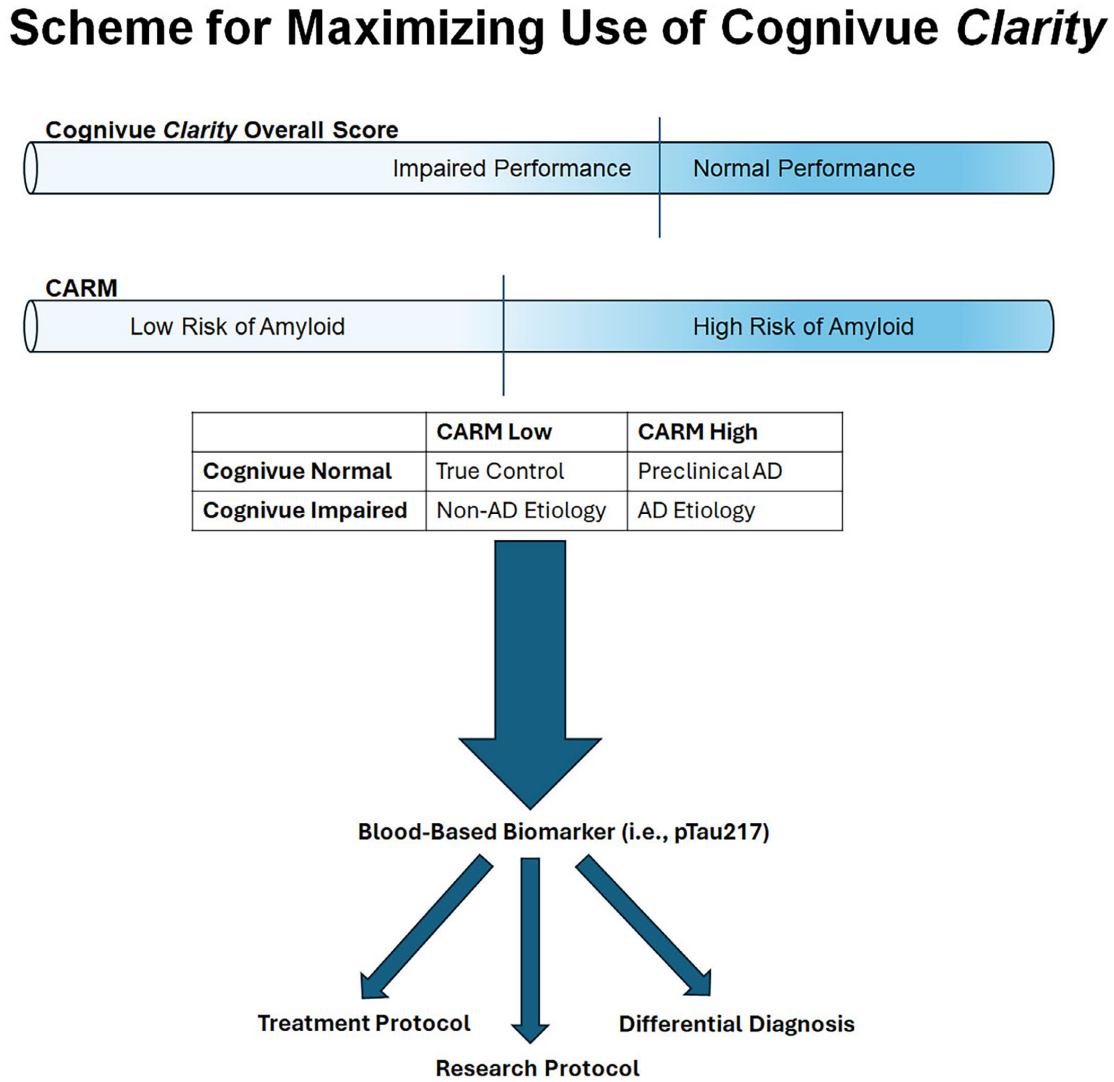# Two Stage Screening for Alzheimer's Disease Clinical Trial Recrutiment Enrichment: Cognivue Clarity and Plasma pTau217

**DOI:** 10.1002/alz70856_101139

**Published:** 2025-12-25

**Authors:** James E Galvin, Michael J Kleiman, Paul W. Estes, Heather M. Harris, Ernest Fung

**Affiliations:** ^1^ University of Miami Miller School of Medicine, Boca Raton, FL, USA; ^2^ Cognivue, Inc., Victor, NY, USA

## Abstract

**Background:**

Clinical detection of amyloid‐positive individuals is generally not possible without expensive biomarkers. This results in delays in diagnosis of Alzheimer's disease (AD) and Mild Cognitive Impairment due to AD (MCI‐AD) reducing the window for treatment with amyloid‐lowering therapies and missed opportunities for enrollment into clinical trials.

**Method:**

887 individuals in the Bio‐Hermes Study (Global Alzheimer's Platform Foundation) completed Cognivue Clarity, amyloid PET, and pTau217. The 4‐level Cognivue Amyloid Risk Measure (CARM) was derived using a machine learning paradigm. We developed a rapid screening paradigm for MCI‐AD and AD.

**Result:**

The cohort had a mean age of 71.8 ± 6.7y, 15.5 ± 2.7y of education, 56.4% female, 37.3% ApoE e4 carriers, and was 78.8% non‐Hispanic White. The clinical‐pathological diagnoses were 297 True Controls, 91 Preclinical AD, 111 MCI‐AD, 171 MCI‐non‐AD, 130 AD dementia, and 87 non‐AD dementia. Amyloid PET SUVR (*p* <.001) and pTau217 (*p* <.001) levels were significantly different by Cognivue Clarity thresholds. Amyloid positivity increased across the 4 CARM thresholds (*p* <.001). Combining Cognivue Clarity global scores and CARM created a 2x2 paradigm of Impaired/Not Impaired, and Low/High risk of amyloid with significant differences in Amyloid PET SUVR (*p* <.001) and pTau217 (*p* <.001). The majority of MCI‐AD, and AD dementia individuals were in the Cognivue Impaired, CARM 3/4 category. Most True Controls were in the Cognivue Not‐Impaired, CARM 1/2 category. Non‐AD cases were scattered across all 4 categories. Preclinical AD individuals had lower Cognivue Clarity global scores than True Controls (*p* <.001) and were largely CARM 3/4.

**Conclusion:**

Cognivue *Clarity*, a 10‐minute computerized battery, can detect individuals with cognitive impairment and with the CARM can identify individuals likely to have amyloid positivity. To further increase the efficiency and cost‐effectiveness of cognitive screening, a staged screening approach likely makes the most sense. Cognivue Clarity global scores establish whether there is cognitive impairment, and in the same sitting CARM predicts the likelihood of amyloid. This could be followed by measuring a readily accessible AD biomarker such as plasma pTau217. Such a strategy would increase the likelihood of identifying early AD for treatment or trial enrollment, avoiding the cost of expensive PET scans in a time‐ and cost‐effective fashion.